# Strategies and mechanisms targeting *Enterococcus faecalis* biofilms associated with endodontic infections: a comprehensive review

**DOI:** 10.3389/fcimb.2024.1433313

**Published:** 2024-07-18

**Authors:** Shipeng Yang, Xiuping Meng, Yuqi Zhen, Quzhen Baima, Yu Wang, Xinmiao Jiang, Zhibo Xu

**Affiliations:** ^1^ Department of Endodontics, Hospital of Stomatology, Jilin University, Changchun, China; ^2^ Department of Dental Implantology, Hospital of Stomatology, Jilin University, Changchun, China

**Keywords:** *Enterococcus faecalis*, biofilms, endodontic infection, antibiotic resistance, mechanisms

## Abstract

*Enterococcus faecalis* is one of the main microorganisms that infects root canals, ranking among the most prevalent microorganisms associated with endodontic treatment failure. Given its pervasive presence in persistent endodontic infections, the successful elimination of *Enterococcus faecalis* is crucial for effective endodontic treatment and retreatment. Furthermore, *Enterococcus faecalis* can form biofilms - defense structures that microbes use to fight environmental threats. These biofilms confer resistance against host immune system attacks and antibiotic interventions. Consequently, the presence of biofilms poses a significant challenge in the complete eradication of *Enterococcus faecalis* and its associated disease. In response, numerous scholars have discovered promising outcomes in addressing *Enterococcus faecalis* biofilms within root canals and undertaken endeavors to explore more efficacious approaches in combating these biofilms. This study provides a comprehensive review of strategies and mechanisms for the removal of *Enterococcus faecalis* biofilms.

## 1 Background

*Enterococcus faecalis* is regarded as the primary cause of persistent infections and failed root canal treatments, its robust survival capabilities under adverse environmental conditions (Siqueira and Rôças, 2022). In particular, *Enterococcus faecalis* exhibits resilience towards elevated pH levels, enabling it to thrive in the presence of calcium hydroxide (Ca(OH)_2_) commonly employed during root canal treatment (Elashiry et al., 2023). This adaptability can be attributed to the utilization of a proton pump by *Enterococcus faecalis* within their plasma membrane, facilitating effective regulation of cytoplasmic equilibrium and endurance against high alkalinity (Divakar et al., 2020). Therefore, in the presence of this bacterium within an infected root canal, utmost efforts should be made to eliminate it. It is important to note that *Enterococcus faecalis* has the ability to form biofilms. The presence of these biofilms creates a protective milieu that diminishes the efficacy of the host’s immune system and antibiotics (Ramakrishnan et al., 2022). Successful root canal treatment depends on preventing and eliminating the biofilms (Abusrewil et al., 2020). In clinical, sodium hypochlorite (NaOCl) and chlorhexidine (CHX) are commonly employed as irrigating solutions for biofilm removal. NaOCl is utilized in various concentrations due to its antibacterial properties and ability to dissolve organic tissue; however, it is important to consider its cytotoxicity and potential irritation in the periapical region (Haapasalo et al., 2014). On the other hand, CHX exhibits broad-spectrum antibacterial activity with lower toxicity compared to NaOCl (Ruksakiet et al., 2020). Nevertheless, it lacks the capability to dissolve organic tissue (Gomes et al., 2013). Despite their widespread use, both NaOCl and CHX have limitations that can impact the overall success of root canal treatments. Consequently, there is increasing interest in developing new methods to enhance the efficacy of biofilm removal. This article provides a comprehensive review of recent advancements in research on strategies and mechanisms targeting *Enterococcus faecalis* biofilms.

## 2 Enterococcus faecalis and biofilms

### 2.1 Enterococcus faecalis

*Enterococcus faecalis*, a gram-positive anaerobic bacterium originating from commensal bacteria, stands out as one of the most formidable infectious pathogens (Salem et al., 2022). It has been implicated in a spectrum of life-threatening diseases, including infective endocarditis (Pericas et al., 2014; Koehler et al., 2019), urinary tract infection (UTI) (Tebruegge et al., 2011; Li et al., 2020), and meningitis (Tebruegge et al., 2011; Cournac et al., 2012), etc. *Enterococcus faecalis* is commonly detected in instances of persistent or secondary endodontic infections (Gaeta et al., 2023). A study using Polymerase Chain Reaction analysis revealed a detection rate of 79.5% for *Enterococcus faecalis*, with 67.5% of primary infections and 89.6% of secondary infections in root canal (Sedgley et al., 2006). It is obvious that the detection rate of *Enterococcus faecalis* in secondary infections is significantly higher than that in primary infections. Furthermore, it demonstrates resilience to various substances, including drugs like Ca(OH)_2_, flushing agents such as NaOCl, and adverse conditions, including low pH, high salinity, and elevated temperatures (Pinheiro et al., 2003; Narayanan and Vaishnavi, 2010). Accordingly, *Enterococcus faecalis* should be regarded as a primary target for therapeutic strategies in the context of root canal infection treatment or retreatment (Elashiry et al., 2023).

### 2.2 Biofilms

**Figure 1:**
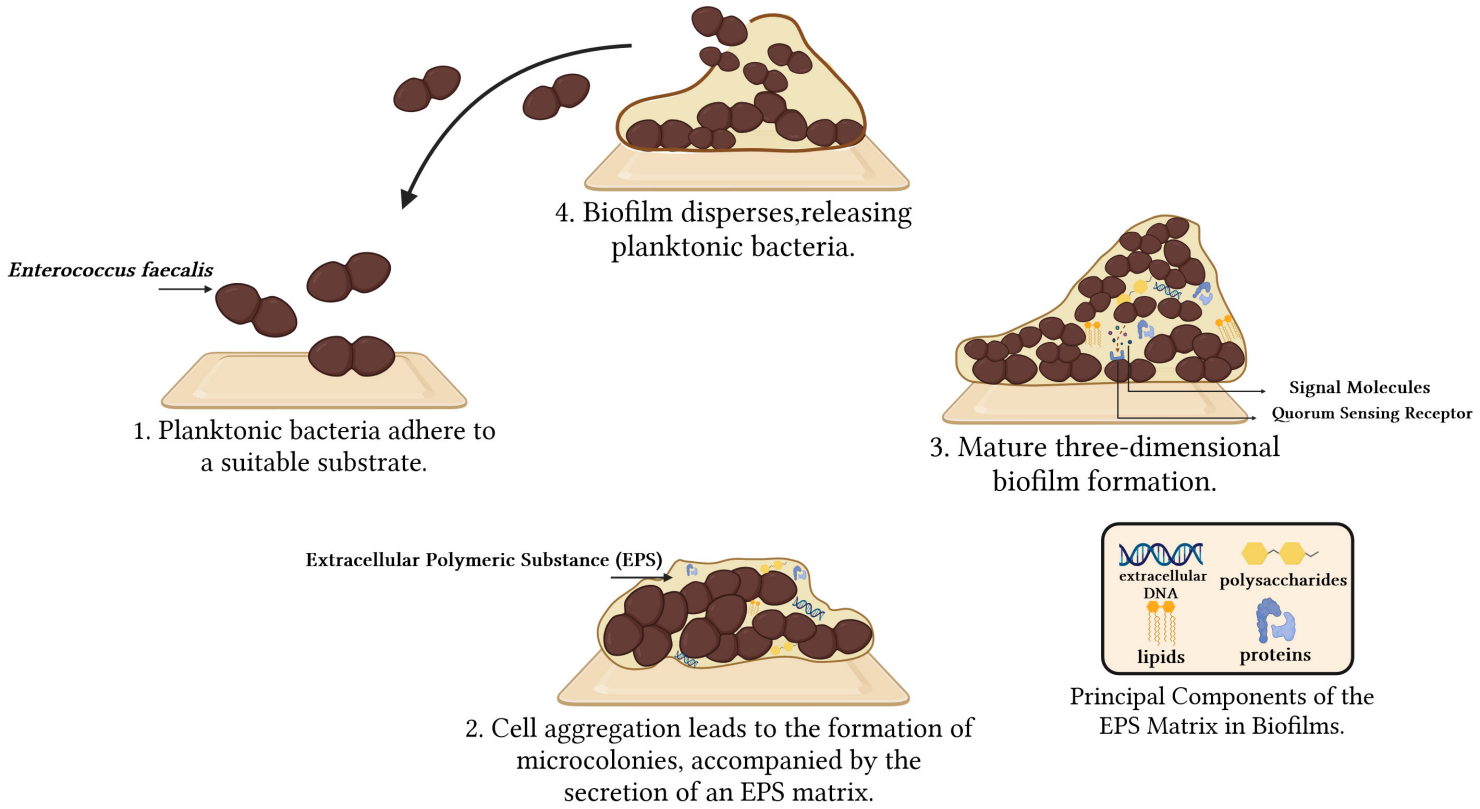
From adherence to dispersal: Four Phases of *Enterococcus faecalis* biofilm formation.

Biofilms represent self-synthesizing layers of intricate microbial communities composed of extracellular polymeric substance (EPS), encompassing polysaccharides, proteins, lipids, and extracellular DNA (eDNA). The EPS typically exhibits a thickness ranging from 0.2 to 1.0 μm, with the overall size of the biofilms remaining within the range of 10 to 30 nm (Jamal et al., 2018). Biofilms provide several benefits for *Enterococcus faecalis*. They establish a protective layer that defends against external threats, such as antibiotics and immune system attacks. Moreover, biofilms promote bacterial growth and reproduction, enhance bacterial viability by facilitating the exchange of nutrients and signaling molecules, assist in bacterial attachment and localization in specific environments, and optimize bacterial resource utilization and adaptation (Karatan and Watnick, 2009). Biofilms can significantly increase the necessary dosages of certain antibiotics by up to 1000 times to combat infections caused by them (Ramakrishnan et al., 2022). Biofilms development encompasses four key stages: 1) Planktonic bacteria adhere to a suitable substratum; 2) As adherent cells irreversibly attach to the surface, they form microcolonies and secrete an EPS matrix. This irreversible attachment is crucial as it marks the transition from a reversible to a stable state, anchoring the cells firmly to the surface; 3) The biofilms matures through the development of microcolonies and the establishment of a water channel architecture. It undergoes a notable increase in layering, and upon reaching full maturation, it achieves peak cell density, ultimately transforming into a three-dimensional community partially regulated by quorum sensing (QS); 4) The mature biofilms release microcolonies of cells from the primary community, allowing them to freely migrate to new surfaces and propagate the infection to other locations (Ch'ng et al., 2019; Verderosa et al., 2019; Ramakrishnan et al., 2022) (**Figure 1**). During the biofilm formation process, numerous genes are activated, including those that govern surface adhesion, aggregation, extracellular polymorphism, and toxin production. Consequently, these gene products enhance the development and colonization of biofilms, ultimately increasing adhesion to host tissues (Anderson et al., 2015). Biofilm formation is primarily controlled genetically through three mechanisms: quorum sensing, cyclic dinucleotide signaling, and small non-coding RNAs (sRNAs). Only a few studies have been conducted on cyclic dinucleotides and sRNAs, while the majority of studies have focused on the QS system (Kalia et al., 2023).

### 2.3 *Enterococcus faecalis* biofilms

The ability of *Enterococcus faecalis* to withstand starvation and form biofilms has been demonstrated across various environmental and nutritional conditions, including aerobic, anaerobic, nutrient-rich, and nutrient-deprived environments (Jhajharia et al., 2015). These biofilms strongly adhere to areas beyond the primary root canals, including apical deltas, isthmuses, and lateral canals. This attachment leads to a reduction in antimicrobial efficacy due to the presence of tissue residues, serum, dead cells, and dentin (Prada et al., 2019).

*Enterococcus faecalis* biofilms exhibit physicochemical properties that dynamically vary in response to the surrounding environmental and nutritional conditions. In a nutrient-rich environment, they produced typical biofilm structures with characteristic surface aggregates of bacterial cells and water channels. In contrast, under a nutrient-deprived environment, irregular growth of adherent cell clumps was observed (Jhajharia et al., 2015). During starvation, cells grown in biofilms are characterized by increased protein synthesis and decreased nucleic acid levels (Deng et al., 2023). The formation of *Enterococcus faecalis* biofilms involves an intricate interplay among secreted virulence factors, surface proteins, quorum-sensing molecules, and regulators governing the release of eDNA. They are involved in bacterial adhesion, biofilm formation, resistance to killing, and tissue damage. Gelatinase (GelE), a crucial virulence factor encoded by the gelE gene, is responsible for collagen and other proteins degradation, while also facilitating the release of eDNA by activating the major autolysin AtlA. Cytolysin, another significant factor, contributes to biofilm formation by lysing other bacterial cells and releasing eDNA, thereby promoting bacterial aggregation and stability within the biofilm matrix. Surface proteins, such as pili and microbial surface components recognizing adhesive matrix molecules, including Ace, play pivotal roles in bacterial adhesion to host tissues and abiotic surfaces. The QS system in *Enterococcus faecalis*, particularly the fsr operon, governs the transcriptional regulation of gelatinase and serine protease, thereby exerting a significant influence on biofilm formation (Schiopu et al., 2023). Understanding these mechanisms lays the foundation for developing targeted anti-biofilm therapies.

**Figure 2:**
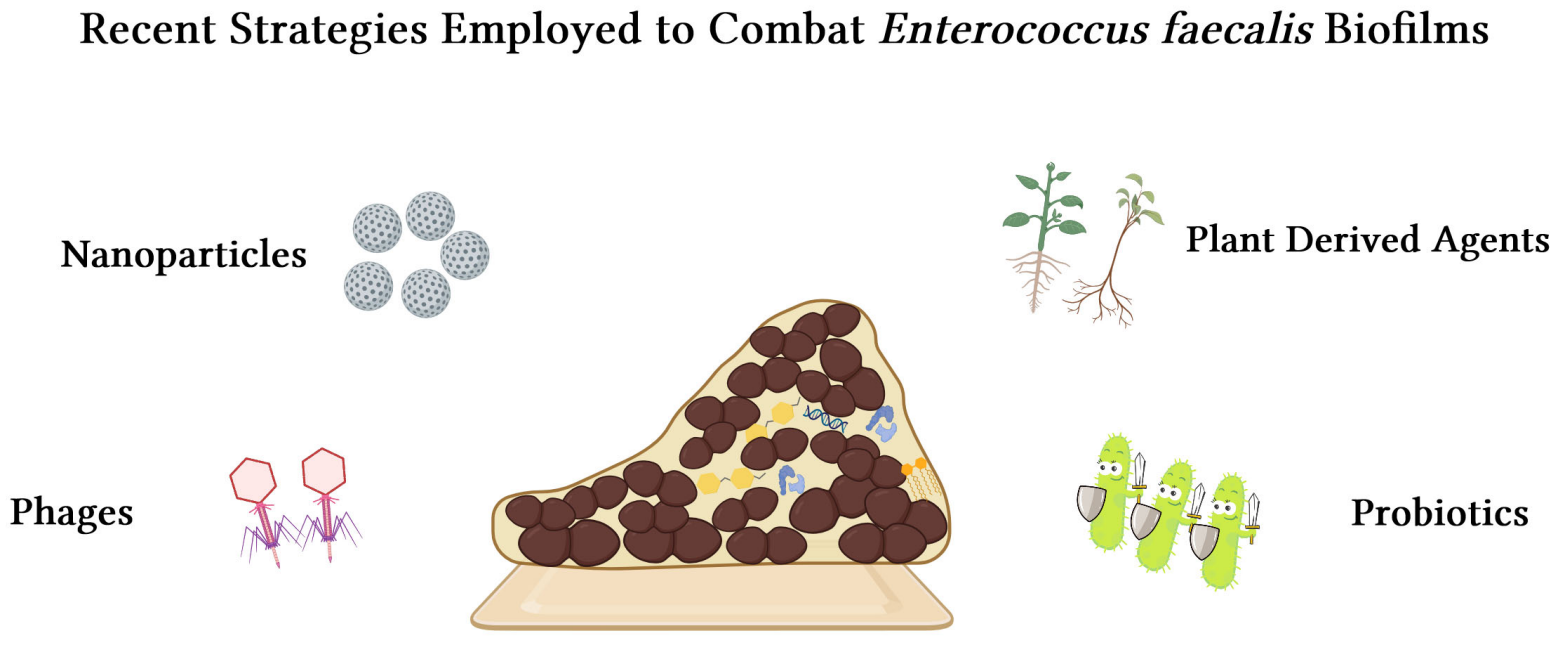
Merging strategies to combat *Enterococcus faecalis* biofilms: nanoparticles, phages, plant derived agents, and probiotics.

Moreover, within the biofilms, *Enterococcus faecalis* engages in intricate interactions with a diverse array of microorganisms to foster the genesis and fortitude of biofilms. For example, the gelatinase of *Enterococcus faecalis* enhances the cross-feeding of heme by extracting it from *Staphylococcus aureus* hemoproteins, thereby promoting the growth of dual-species biofilms (Ch'ng et al., 2022). In the initial stages of biofilm formation, AI-2 produced by *Enterococcus faecalis*, which acts as a chemoattractant, facilitates rapid auto-aggregation and growth of *Escherichia coli*, thereby enhancing mixed-species biofilms’ resilience against hostile stressors (Laganenka and Sourjik, 2018). *Enterococcus faecalis* also stimulates the synthesis of exopolysaccharides in *Pseudomonas aeruginosa*, thus promoting biofilm growth and biomass by inducing the expression of Pel and Psl (Lee et al., 2017). The formation of *Enterococcus faecalis* biofilms enhances bacterial resistance against antibiotics (Salem et al., 2022), evades host immune responses (Fleming and Rumbaugh, 2017). The conventional mechanical preparations and irrigation in root canal are insufficient for achieving comprehensive clearance, thereby impeding the attainment of desired levels of infection control. Recently, diverse strategies have emerged for addressing infections associated with *Enterococcus faecalis* biofilms. These strategies include employing nanoparticles, phages, plant derived agents, and probiotics against *Enterococcus faecalis* biofilms (**Figure 2**).

## 3 Strategies for combating *Enterococcus faecalis* biofilms

### 3.1 Nanoparticles

Nanoparticles, approximately 1 to 100 nm in size, exhibit unique physicochemical properties due to their ultra-small particle size, high surface area-to-mass ratio, and enhanced chemical reactivity (Afkhami et al., 2024). Specifically, nanoparticles smaller than 5 nm tend to demonstrate increased biocompatibility and are more favorable to biological systems, potentially causing less disruption to normal biological and physiological functions (Xie et al., 2020). Moreover, they possess a unique capability to penetrate tissues, infiltrate bacterial cell walls, interact with biofilms, and engage bacteria (Halkai et al., 2018). It has been documented that nanosilver disrupts bacterial cell membranes and induces the production of reactive oxygen species (ROS), leading to protein denaturation, DNA damage, and interference with respiratory enzymes (Chernousova and Epple, 2013).

Nanoparticles, particularly silver nanoparticles, have been extensively investigated for their potential in root canal disinfection. Nagendrababu et al. demonstrated that silver nanoparticles were more effective in eradicating *Enterococcus faecalis* biofilms than CHX in an *in vitro* study (Nagendrababu et al., 2017). Balto et al. further demonstrated that the combined application of silver nanoparticles and Ca(OH)_2_ remarkably enhanced the eradication of *Enterococcus faecalis* compared to the use of either agent alone. This combination was shown to significantly inhibit biofilm formation, achieving a biofilm inhibition rate of over 90% in an *in vitro* study (Balto et al., 2020). However, it has been reported that silver nanoparticles possess potential adverse effects such as tooth discoloration. Although Afkhami et al. reported no significant alteration in tooth color with the addition of silver nanoparticles compared to Ca(OH)_2_ alone, and extending the application time to 3 months did not result in an increase in color change, there is still an ongoing controversy regarding their long-term use as root canal medications *in vivo* (Afkhami et al., 2017). Therefore, further investigation is imperative to comprehensively explore any potential adverse effects in this scenario.

Apart from silver nanoparticles, numerous other nanoparticle materials have demonstrated efficacy in combating *Enterococcus faecalis* biofilms. Arias-Moliz et al. synthesized doxycycline-functionalized polymeric nanoparticles, which not only inhibit the formation of biofilms by *Enterococcus faecalis* on dentin but also exhibit the capability to disrupt pre-formed biofilms. Furthermore, these nanoparticles were observed to reduce bacterial invasion into occluded dentinal tubules by competing with bacteria for that specific site. Moreover, it has been confirmed that these materials can bind to the external EPS within *Enterococcus faecalis* biofilms, thereby significantly disrupting their structural organization and overall integrity (Arias-Moliz et al., 2021). Parolia et al. employed the ultrasonication method to prepare propolis nanoparticles and evaluated their effectiveness in eliminating *Enterococcus faecalis* biofilms *in vitro* by scanning electron microscopy (SEM) and confocal laser scanning microscopy (CLSM). They demonstrated that preparing propolis nanoparticles as an endodontic irrigant was equally as effective as 6% NaOCl and 2% CHX in reducing *Enterococcus faecalis* colony forming unit (CFU) in a human tooth model and *Enterococcus faecalis* isolates obtained from patients with failed root canal treatment (Parolia et al., 2021). Parolia A et al. evaluated the antibacterial effect of chitosan-propolis nanoparticle (CPN) as an intracanal medicament against *Enterococcus faecalis* biofilm in root canal, and concluded CPN was more effective in reducing *Enterococcus faecalis* CFU than saline, chitosan, propolis nanoparticles and CHX on day one and day three, and at day seven CPN was equally effective as 2% CHX (Parolia et al., 2020).

Graphene, a nanoparticle material composed of a single layer of carbon atoms, exhibits diverse physical properties including stretchability, electrical conductivity, large surface area, and high thermal conductivity (Martini et al., 2020; Kim et al., 2023). The synthesis of graphene on a large scale poses challenges, and researchers have explored derivatives of graphene that are more suitable for large-scale synthesis (Kim et al., 2023), such as graphene oxide (GO) and reduced graphene oxide (RGO). The exceptional physicochemical properties, morphological characteristics, biocompatibility, and antimicrobial activities of both graphene and its derivatives have attracted significant attention in the field of dentistry (Li et al., 2022). In a study conducted by Martini et al., the application of GO as a coating on dentin discs, followed by incubation with *Enterococcus faecalis* for 72 hours, resulted in a significant inhibition of bacterial attachment and biofilm formation (Martini et al., 2020). Kim et al. investigated the impact of GO and RGO on the growth of *Enterococcus faecalis* biofilms on hydroxyapatite discs *in vitro* and found that both GO and RGO effectively inhibited the proliferation of *Enterococcus faecalis*. Meanwhile, they found that RGO exhibited lower cytotoxicity compared to GO (Kim et al., 2023). Consequently, these findings suggest that RGO may possess greater potential as a root canal disinfection agent against *Enterococcus faecalis* biofilms.

The application of low electrical currents has been demonstrated to be a viable and effective method for sterilization, efficiently eliminating bacteria and biofilms (Valle et al., 2007; Minkiewicz-Zochniak et al., 2021). It is attributed to the enhanced detachment of biofilms from the substrate facilitated by electrical energy, likely resulting from increased repulsive effects induced by the current. The study conducted by Lee et al. investigated the effect of electrical energy and GO on *Enterococcus faecalis* biofilms, revealing that their combined application enhances the antimicrobial activity of NaOCl (Lee et al., 2023). These findings highlight the potential applications of this approach in root canal irrigation protocols.

The nanoparticles containing metals, drugs, or natural products used for the treatment of anti-*Enterococcus faecalis* biofilm-mediated infections may offer broader therapeutic potential. However, their safety and effectiveness in addressing biofilm infections require comprehensive testing and evaluation in clinical practice to substantiate their efficacy.

### 3.2 Phages

Phages are viral entities capable of infecting and dismantling bacteria. They possess the unique ability to infiltrate bacterial biofilms and induce damage. The efficacy of phage therapy has been demonstrated to be superior to that of conventional antibiotics, particularly in cases involving infections caused by multi-drug resistant biofilms (Khalifa et al., 2015a; Chan et al., 2018). Phages exhibit specificity towards particular bacterial species or even individual strains, making them an ideal therapeutic option for selectively targeting and eliminating pathogens (Kortright et al., 2019). Typically, phages have a highly favorable safety profile, and phage therapy is generally associated with a low risk of adverse reactions and side effects (Hatfull et al., 2022).

**Figure 3:**
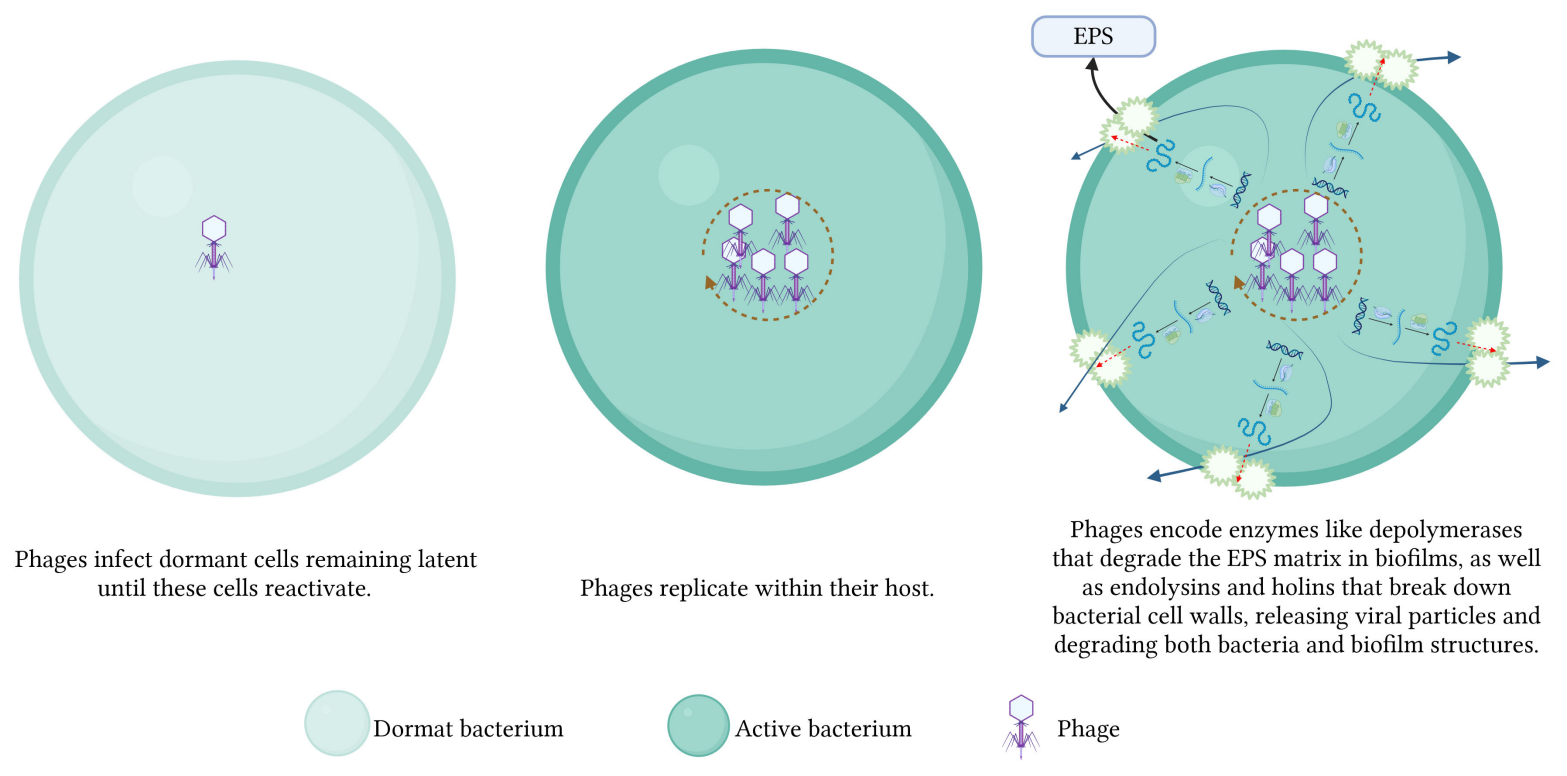
Mechanisms of phage-mediated biofilm disruption - part 1.

**Figure 4:**
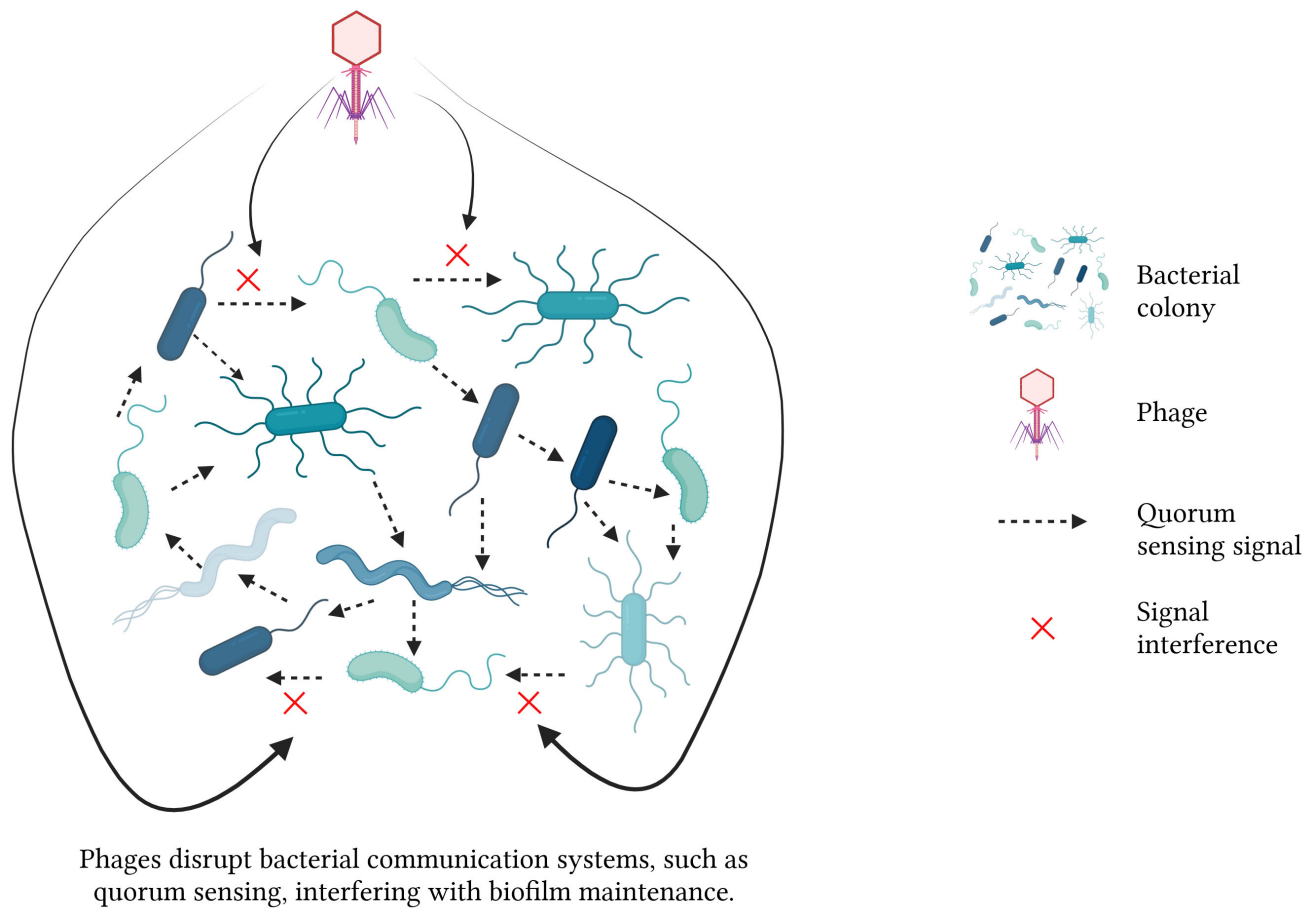
Mechanisms of phage-mediated biofilm disruption - part 2.

Phages disrupt bacterial biofilms through a multitude of intricate mechanisms. 1) The phages replicate within bacterial cells, resulting in cell lysis and the subsequent release of progeny phages that gradually dismantle the biofilms. 2) Some phages infect dormant cells, remaining latent until these cells reactivate, subsequently lysing them. 3) Phages encode enzymes like endolysins and holins that degrade bacterial cell walls, facilitating the release of viral particles and the breakdown of biofilm structures. Additionally, phage-produced depolymerases degrade the EPS matrix in biofilms, destabilizing the biofilm and allowing for deeper penetration and more effective eradication (**Figure 3**). 4) Phages disrupt bacterial communication systems, such as QS, thereby interfering with the regulatory processes critical for biofilm maintenance and stability (Khalifa et al., 2016; Moryl et al., 2023) (**Figure 4**). These combined mechanisms highlight the potential of phage therapy in effectively targeting and eradicating biofilms.

To combat *Enterococcus faecalis* infections, more than 25 phages have been isolated; most of them belong to the Myoviridae or Siphoviridae families of tailed phages. Among these, recently phages EFDG1 and EFLK1 have been investigated for their efficacy against *Enterococcus faecalis* biofilm formation. Khalifa et al. discovered that phage EFDG1, isolated from sewage effluent, exhibits significant lytic activity against *Enterococcus faecalis*. It was confirmed that phage EFDG1 can effectively eliminate 100 mm thick biofilm existing for 2 weeks formed by *Enterococcus faecalis* V583 (Khalifa et al., 2015a). However, resistance to phage EFDG1 has emerged in *Enterococcus faecalis* strains. To specifically target these resistant strains, Khalifa et al. successfully isolated a novel phage designated as EFLK1 (Khalifa et al., 2015b). The efficacy of this phage against both the resistant mutant strain EFDG1r and its parental strain, vancomycin-resistant Enterococcus (VRE)-*Enterococcus faecalis* V583, was demonstrated (Khalifa et al., 2018). The study conducted by Shlezinger et al. demonstrated that treatment with EFLK1 effectively disrupts and diminishes biofilm formation, resulting in a significant reduction of approximately 81% (Shlezinger et al., 2019). Engineered bacteriophage phiEf11 (phiEf11/phiFL1C(Δ36)) ^PnisA^ reduced the static biofilm formed by *Enterococcus faecalis* strains JH2-2 (pMSP3535nisR/K) and V583 (pMSP3535nisR/K) by 10–100 times after cultivation for 24–48 hours (Tinoco et al., 2016). *Enterococcus faecalis* phage SHEF2, isolated from the mouth of a patient with an infected root canal, could eradicate the biofilm formed on the surface of polystyrene *in vitro*, and resolve an *Enterococcus faecalis* infection in a zebrafish model system. It can be used to treat antibiotic-resistant *Enterococcus faecalis* infection (Al-Zubidi et al., 2019). Compared with antibiotics, phages generally have a narrower inhibition spectrum and stronger targeting ability. Studies in a mouse model have indicated that combined therapy of phages and the antibiotic ampicillin notably enhances the efficacy against Vancomycin-Resistant *Enterococcus faecalis* (Gelman et al., 2018). When used in combination with vancomycin, the biomass of *Enterococcus faecalis* biofilms decreased by 87%, demonstrating a synergistic effect of phages in disrupting and reducing biofilm formation (Shlezinger et al., 2019). Additionally, Song et al. studied the bacteriophage vB_EfaM_LG1 (LG1), isolated from hospital sewage, and discovered that its combination with an antibiotic significantly inhibited and degraded *Enterococcus faecalis* biofilms (Song et al., 2021).

However, phage therapy also encounters a significant challenge of bacterial resistance to both antibiotics and phages. Phages play an important role in horizontal gene transfer among bacterial populations and may lead to the transfer of antibiotic resistance genes (Yang et al., 2024). Additionally, what is noteworthy is bacteria have developed various mechanisms to evade phage infections. Random mutations or phenotypical variations in bacteria result in decreased phage adsorption. Host defense systems evolved in bacteria to prevent or suppress phage infections. Phage-derived defense systems enable them to compete with other phages, ultimately benefiting the host bacterium (Egido et al., 2022). To enhance the efficacy of phage therapy and mitigate bacterial resistance, several strategies have been proposed. The first approach involves enhancing the bactericidal efficacy of phages by increasing their dosage and utilizing highly lytic phages, which can rapidly diminish bacterial populations and minimize the likelihood of resistance mutations emerging. Another strategy involves utilizing broad-spectrum and potent phages, either by selecting phages capable of infecting a wide range of bacterial strains or using phage cocktails, which significantly lowers the chances of bacteria developing resistance. Combining phages with antibiotics can also reduce the development of phage resistance while enhancing overall antimicrobial efficacy. Additionally, experimental evolution of phages through laboratory-guided co-evolution of phages and bacteria can lead to the development of “trained” phages that are more effective in combating resistant bacterial strains (Torres-Barcelo et al., 2022).

Phage therapy presents a promising alternative to conventional antibiotics combating *Enterococcus faecalis*, however, its successful integration into clinical practice necessitates addressing inherent limitations through ongoing research and technological advancements. Future research should prioritize elucidating phage-bacteria interactions at the molecular level, optimizing phage production and delivery systems, as well as establishing robust regulatory frameworks to ensure the safety and efficacy of phage-based treatments. Additionally, exploring the combined use of phage therapy with traditional antibiotics or novel antimicrobial agents could help mitigate the risk of resistance development. By navigating these challenges, phage therapy has the potential to revolutionize the treatment of multidrug-resistant infections and biofilm-related diseases.

### 3.3 Plant derived agents

In recent years, an increasing body of scholarly research has revealed the potential of various novel plant derived agents in combating *Enterococcus faecalis* biofilms.

#### 3.3.1 Phytotherapeutic agents

##### 3.3.1.1 Trans-cinnamaldehyde

Trans-Cinnamaldehyde (TC) is extracted from the bark of the cinnamon tree, which contains a significantly high concentration of TC, typically ranging between 65% and 80% (Yanakiev, 2020). Extensive research has focused on TC and its derivatives, revealing their notable antimicrobial efficacy against various pathogenic fungi and molds (Doyle and Stephens, 2019).

In their study, Ali et al. made the discovery that TC exhibited comparable efficacy to 1% NaOCl and 2% CHX in eradicating *Enterococcus faecalis* biofilms on dentin discs within a 15-minute timeframe. Notably, TC demonstrated persistent antimicrobial effects on *Enterococcus faecalis* biofilms, inhibiting regrowth even under favorable growth conditions, while viable *Enterococcus faecalis* were observed ten days after treatment with CHX (Ali et al., 2020a). These findings highlight its potential as an effective agent for managing *Enterococcus faecalis* infections during root canal treatments.

However, the biological efficacy of the phytochemical TC is limited by its inherent lipophilicity, which diminishes its solubility in aqueous environments, particularly within the hydrated EPS matrix (Li et al., 2016; Ali et al., 2020b). This limitation may potentially impede the effectiveness of TC in inhibiting biofilm formation. To address this challenge, Hu et al. employed acidic sophorolipids (ASL) as a surfactant to encapsulate TC molecules and form a complex aimed at augmenting the antimicrobial and anti-biofilm properties of hydrophobic trans-cinnamaldehyde. Their findings demonstrated that the TC-ASL complex significantly enhanced bactericidal effects, resulting in a substantial reduction in both biomass and volume of *Enterococcus faecalis* biofilms compared to using TC alone (Hu et al., 2022).

There is evidence indicating the susceptibility of TC to degradation upon exposure to air or blood vessels, resulting in its conversion into cinnamic acid and subsequent reduction in antimicrobial activity (Vasconcelos et al., 2018). Therefore, it is crucial to evaluate its clinical significance, necessitating further investigation into its *in vivo* roles and effects. Addressing the inherent instability of this lipophilic compound and its limited biological activity may require the discovery or synthesis of stable derivatives of TC. In future research, exploring diverse adjuvants such as surfactants, nanomaterials, and other alternatives could significantly contribute to enhancing the solubility and permeability of TC.

##### 3.3.1.2 Quercetin

Quercetin, a naturally occurring flavonoid found in various fruits and vegetables, is renowned for its diverse pharmacological effects, including antioxidant, anti-inflammatory, and anti-aging properties (Wang et al., 2021). Flavonoids, one of the largest groups of polyphenols, possess potent inhibitory properties against viruses, bacteria, and fungi (Cushnie and Lamb, 2005). Quercetin has extensive applications in the treatment of various diseases such as diabetes, obesity, circulatory dysfunction, inflammation, and mood disorders. Furthermore, it exhibits remarkable antitumor, viral, and bacterial activities (D'Andrea, 2015). Qayyum et al. discovered that quercetin demonstrated significant inhibitory effects against *Enterococcus faecalis*, with the lowest inhibitory concentration recorded at 512 mg/L (Qayyum et al., 2019). At this concentration, quercetin exhibited a 95% inhibition rate on biofilm formation. Confirmatory evidence through SEM and CLSM supported the inhibitory impact of quercetin on *Enterococcus faecalis* biofilm formation. Considering the favorable safety profile of quercetin, it appears feasible to conduct *in vivo* studies for validating its clinical relevance (Bischoff, 2008).

#### 3.3.2 Plant extract

##### 3.3.2.1 Grape seed extract

Grape seed extract (GSE) exhibits antioxidant, anticancer, anti-inflammatory, and antimicrobial properties, showcasing its potential for controlling and preventing oral microorganisms (Karygianni et al., 2015). The grape seed extract contains proanthocyanidins (PAs), also referred to as tannins, constituting 2.9% of the extract composition. PAs represent a class of phenolic compounds (Soetanto et al., 2019). It effectively eliminates Streptococcus mutans and serves as a preventive measure against oral diseases such as periodontitis (Mageshwaran et al., 2012). Soetanto et al. employed CLSM analysis to evaluate the antimicrobial impact of a 6.5% concentration of GSE on *Enterococcus faecalis* biofilms (Fiallos et al., 2020). The findings revealed that GSE exhibits superior antimicrobial efficacy against *Enterococcus faecalis* biofilms than 2% CHX and saline solution, but it showed a slightly inferior antimicrobial effect to 5.25% NaOCl. However, the application of NaOCl led to a decrease in the strength and resilience of dentin (Cecchin et al., 2015), due to the damage inflicted on both the organic and inorganic components, particularly collagen within dentin (Gu et al., 2017). On the contrary, when used as an irrigant solution, GSE does not compromise the mechanical properties of dentin. Furthermore, GSE demonstrates potential in enhancing tissue stability against collagenase degradation for the treatment of demineralized dentin (Fiallos et al., 2020), and demonstrating low toxicity towards human cells (Katsuda et al., 2015). These findings position GSE as a promising material for root canal irrigation. Future research should further explore its clinical applications and investigate the synergistic antimicrobial effects of combined usage with NaOCl.

##### 3.3.2.2 Tea tree oil

Tea tree oil (TTO) is derived from the leaves of Melaleuca alternifolia (Qi et al., 2021). TTO is extensively used as a safe, natural, and efficient preservative with potent antimicrobial and antifungal properties that cover a broad range of bioactivity and biosafety (Sharifi-Rad et al., 2017). In recent years, TTO has gained attention for its potential in treating various oral conditions. In the context of combating *Enterococcus faecalis* biofilms, Qi et al. investigated the impact of varying concentrations of TTO on *Enterococcus faecalis* biofilm formation. The results demonstrated that TTO concentrations exceeding 0.25% effectively inhibited biofilm development and eradicated established *Enterococcus faecalis* biofilms (Qi et al., 2021).

##### 3.3.2.3 Berberine hydrochloride

Berberine hydrochloride (BBH), a prominent alkaloid derived from *Rhizoma Coptidis*, possesses diverse medicinal properties (Chen et al., 2016), including antimicrobial activity (Bandyopadhyay et al., 2013) and anticancer effects (Wang et al., 2015), among others. Chen et al. discovered that BBH effectively inhibits the formation of *Enterococcus faecalis* biofilms and promotes their dispersion by suppressing the mRNA expression of Sortase A and EPS (Chen et al., 2016). These findings underscore the significant potential of BBH as an adjunctive therapeutic agent for preventing biofilm-related infections.

##### 3.3.2.4 Propolis

Propolis is produced by honeybees through mixing the secretions of their hypopharyngeal glands with the digested product of resins collected from plants (Skoskiewicz-Malinowska et al., 2017). Propolis contains important pharmacologically active ingredients such as flavonoids, which can act on bacterial microbial membranes or cell walls and cause functional and structural damage (Parolia et al., 2021). A comparative evaluation of the efficacy of propolis and Ca(OH)_2_ as root canal drugs for *Enterococcus faecalis* showed that propolis has antibacterial activity comparable to Ca(OH)_2_ (Shamma et al., 2023).

##### 3.3.2.5 Aloe vera

Aloe leaves are from the Liliaceae family, and their appearance resembles a cactus plant. Hypothetically, they contain approximately 75 active components, such as vitamins, enzymes, sugars, minerals, lignin, saponins, amino acids, and salicylic acids. These components exhibit significant anti-inflammatory, antibacterial, antiviral and antifungal properties along with positive hypoglycemic effects. *In vitro* studies have shown that *Aloe vera* exhibits antibacterial capabilities against *Enterococcus faecalis*, which are superior or equal to those of saline and Ca(OH)_2_, but inferior to CHX, NaOCl and propolis (Tariq et al., 2023). Furthermore, Ghasemi et al. found *in vitro* that *Aloe vera* exhibited more significant antibacterial activity against *Enterococcus faecalis* biofilms compared to Ca(OH)_2_ (Ghasemi et al., 2020). These findings suggest that *Aloe vera* has potential as a natural intracanal medicament for combating *Enterococcus faecalis* biofilms in endodontic treatments.

##### 3.3.2.6 Triphala

Triphala is composed of the myrobalans *Terminalia chebula*, *Terminalia bellerica*, and *Emblica officinalis*. It contains tannins, polyphenols, and vitamin C, which confer significant antioxidant, anti-inflammatory, and antimicrobial properties. In dentistry, it serves as an effective endodontic irrigant and periodontal treatment due to its ability to inhibit oral pathogens and promote oral health (Mohanty et al., 2019). Triphala demonstrated significant antimicrobial efficacy against *Enterococcus faecalis* biofilm, although it was less effective compared to NaOCl (Durga Bhavani et al., 2023). In contrast to NaOCl, Triphala offers lower toxicity, ease of availability, cost-effectiveness, and additional health benefits like antioxidant and anti-inflammatory properties (Prabhakar et al., 2010). These advantages make Triphala a promising alternative for root canal irrigation.

##### 3.3.2.7 Other plant extracts

Other plant extracts, such as *Uncaria tomentosa*, apple vinegar, *Zingiber officinale*, castor oil detergent, and essential oils, have also demonstrated pharmaceutical potential against *Enterococcus faecalis* in ex vivo studies.

*Uncaria tomentosa*, known as “cat’s claw”, has proved less effective than CHX against *Enterococcus faecalis*, but it does have anti-inflammatory and immuno-modulating properties. Apple vinegar has demonstrated an 87% reduction in smear layer in *in vitro* experiments, closely matching the efficacy of NaOCl (90%) and Ethylene diamine tetraacetic acid (92%), without causing significant tissue damage or toxicity. The effect of Ginger extracts on *Enterococcus faecalis* was statistically similar to 2.5% NaOCl and 2% CHX in ex vivo studies. The research on the effects of essential oils on *Enterococcus faecalis* is still scarce (Borzini et al., 2016). These studies currently focus solely on the planktonic form of *Enterococcus faecalis*, with less attention to its biofilm state, which plays a critical role in persistent infections. Future research should explore these extracts’ effects on *Enterococcus faecalis* biofilms to better understand their potential in treating biofilm-associated infections.

The aforementioned studies investigating the effects of plant derived agents on *Enterococcus faecalis* were all conducted *in vitro*. These studies demonstrate the promising potential of plant derived agents in combating *Enterococcus faecalis* biofilms, indicating their possible utility in treating persistent endodontic infections caused by *Enterococcus faecalis* biofilms. Future efforts should prioritize evaluating their biosafety and exploring the clinical efficacy of plant derived agents in eradicating *Enterococcus faecalis* biofilms from root canals.

### 3.4 Probiotics

Probiotics are a group of live microorganisms that, when consumed in sufficient quantities, can exert beneficial effects on the human body. The majority of strains currently utilized are derived from the gut microbiota of healthy individuals, with *Lactobacillus* and *Bifidobacteria* being the most frequently employed (Gueimonde and Collado, 2012). Probiotics have emerged as a captivating therapeutic avenue due to studies suggesting their intricate association with gut microbiology and potential efficacy in managing digestive and chronic inflammatory disorders (Oelschlaeger, 2010). Moreover, they have gained attention for their prospective therapeutic application in addressing mental health conditions such as anxiety, depression, and autism (Chidambaram et al., 2020). In terms of oral health benefits, probiotics have demonstrated promise in caries prevention (Näse et al., 2001; Stecksen-Blicks et al., 2009), curtailing gingivitis progression (Staab et al., 2009), and managing conditions like periodontitis (Teughels et al., 2013), among others.

Recent investigations have emerged due to persistent root canal infections attributed to *Enterococcus faecalis* biofilms, prompting scholars to explore the potential of probiotics against *Enterococcus faecalis* and its biofilms. Jung et al. revealed that lipoteichoic acid (LTA) produced by *Lactobacillus* demonstrates the capability to inhibit *Enterococcus faecalis* biofilm formation, exerting a sustained inhibitory effect at an early stage. Furthermore, LTA derived not only from *Lactobacillus* but also from various species such as *Lactobacillus acidophilus*, *Lactobacillus casei*, and *Lactobacillus rhamnosus* exhibited similar inhibitory effects against *Enterococcus faecalis* biofilm formation (Jung et al., 2019). Therefore, LTA derived from *Lactobacillus* species stands as a promising anti-biofilm agent, offering potential applications in preventing or treating diseases associated with *Enterococcus faecalis*.

Bohora et al. conducted a study utilizing three probiotics, namely *Lactobacillus plantarum*, *Lactobacillus rhamnosus*, and *Bifidobacterium bifidum*. The study evaluated the inhibitory activity of these probiotics in both planktonic and biofilm stages. In the planktonic stage, these probiotics exhibited inhibitory activity. During the biofilm stage, their research emphasized that incorporating 30% poloxamer 407 (Pluronic F-127) into the De Man, Rogosa and Sharpe medium containing probiotics resulted in all probiotics demonstrating varying degrees of inhibition in *Enterococcus faecalis* biofilm growth. Moreover, this research suggests that poloxamer 407 (Pluronic F-127) holds promise as an effective delivery vehicle for introducing probiotics into the root canal system (Bohora et al., 2019).

Shaaban et al. investigated the impact of supernatants derived from multi-strain probiotics containing *Lactobacillus plantarum*, *Lactobacillus acidophilus*, and *Lactobacillus rhamnosus* on *Enterococcus faecalis* biofilms. The study findings revealed that after 24 hours, the multi-strain probiotics reduced *Enterococcus faecalis* biofilm formation more effectively than Ca(OH)_2_. However, after 7 days, both the multi-strain probiotics and Ca(OH)_2_ exhibited comparable antimicrobial effects by significantly reducing *Enterococcus faecalis* biofilm counts. These results suggest that a longer contact duration may be necessary to achieve more pronounced antimicrobial effects. Nevertheless, further investigations are required to determine the optimal concentration of the multi-strain probiotics (Shaaban et al., 2023).

Safadi et al. investigated the potential of probiotics as an alternative irrigant solution for eradicating *Enterococcus faecalis* biofilms. The study assessed the impact of three specific probiotic strains (*Lactobacillus casei*, *Lactobacillus plantarum*, and *Bacillus coagulans*) on pre-established *Enterococcus faecalis* biofilms. The findings demonstrated that both *Lactobacillus casei* and *Lactobacillus plantarum* efficiently eradicated established *Enterococcus faecalis* biofilms and inhibited their regrowth; however, *Bacillus coagulans* did not exhibit comparable effectiveness (Safadi et al., 2022).

The potential benefits of probiotics for host health and safety are widely acknowledged by experts (Suissa et al., 2022). However, certain studies have indicated potential risks associated with probiotic use in immunocompromised or chronically ill patients, which could potentially lead to conditions such as sepsis or shock (Boyle et al., 2006). Although current research is still in its preliminary stages, it is imperative that future studies prioritize *in vivo* investigations to validate these initial findings. Prior to commencing these *in vivo* studies, rigorous trial designs and comprehensive biosafety assessments are essential to ensure both safety and efficacy. Moreover, to fully harness the therapeutic potential of probiotics, future research should focus on exploring innovative delivery methods, including biocompatible carriers and sustained-release formulations. These advancements are of critical importance for optimizing the therapeutic benefits of probiotics, thereby enhancing their efficacy and safety for broader clinical applications.

## 4 Mechanisms targeting *Enterococcus faecalis* biofilms

**Figure 5:**
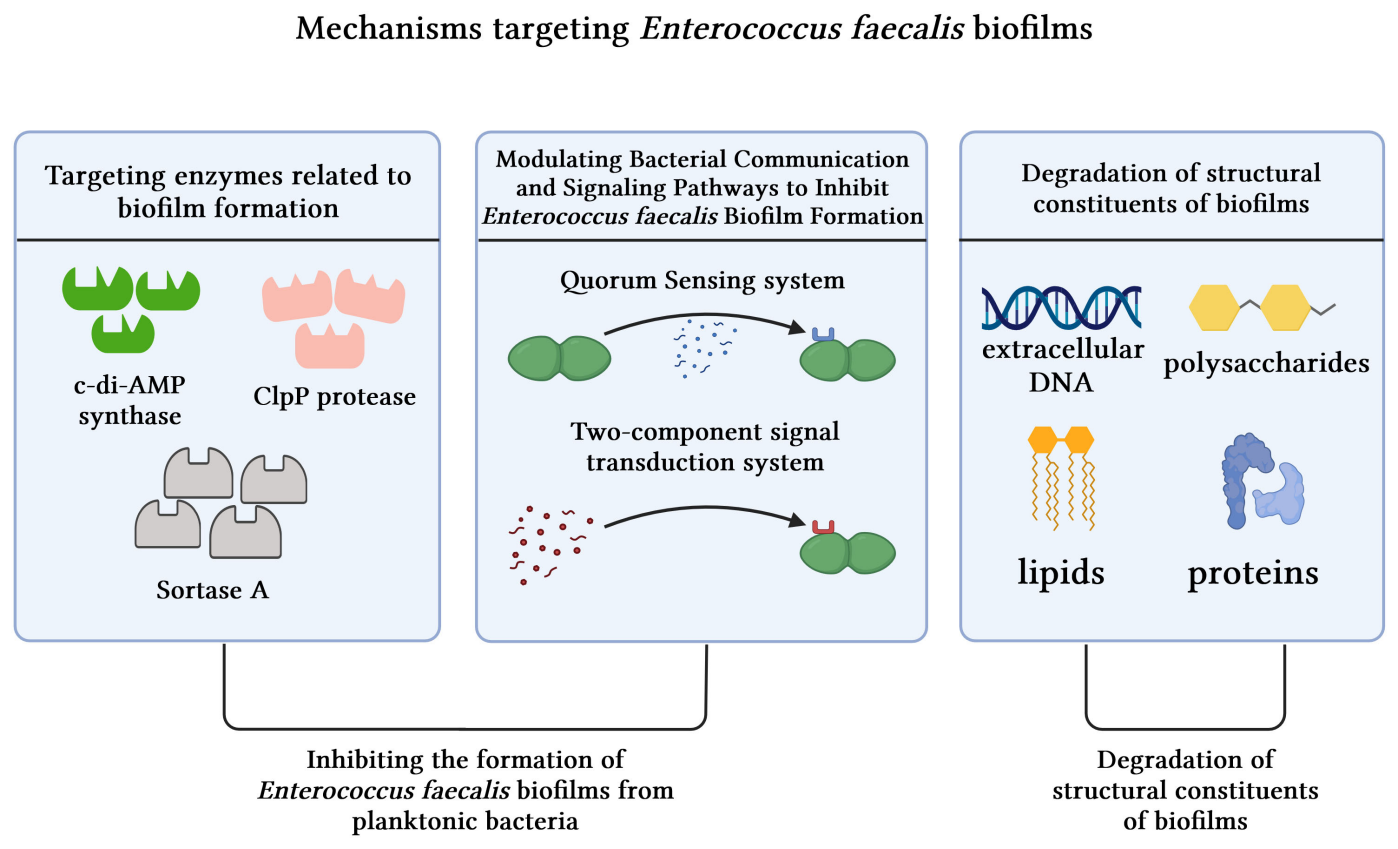
Mechanisms targeting *Enterococcus faecalis* biofilms.

In general, the combat against biofilms involves inhibiting their formation during the developmental process or disrupting pre-formed ones. These mechanisms can be broadly classified into three categories: targeting enzymes associated with biofilm formation, modulating QS and signal transduction systems, and degrading structural components linked to biofilms (**Figure 5**).

### 4.1 Inhibiting the formation of *Enterococcus faecalis* biofilms from planktonic bacteria

#### 4.1.1 Targeting enzymes related to biofilm formation

Biofilm formation represents a multifaceted process reliant on various synthetic enzymes, including c-di-AMP synthase and ClpP protease, among others.

The function of c-di-AMP synthase involves synthesizing c-di-AMP, which is a pivotal signaling molecule within the cell. This molecule significantly influences various essential cellular functions, such as bacterial proliferation, virulence, biofilm production, regulation of cellular morphology, fatty acid synthesis, and modulation of the host immune response (Sureka et al., 2014). In their study, Chen et al. employed sequence alignment and molecular docking techniques to investigate the potential impact of a small molecule known as ST056083 on c-di-AMP synthase in *Enterococcus faecalis*. Furthermore, they conducted a series of phenotypic assays to validate the inhibitory effect of ST056083 on *Enterococcus faecalis* growth and biofilm formation. The experimental findings demonstrate that ST056083 selectively targets the activity of c-di-AMP synthase, thereby effectively suppressing *Enterococcus faecalis* biofilm formation (Chen et al., 2018).

The absence of ClpP protease induces alterations in the growth pattern of *Enterococcus faecalis* and diminishes the polysaccharide matrix, thereby impairing its ability to form biofilms. This suggests a potential therapeutic avenue targeting ClpP protease for the treatment of *Enterococcus faecalis* infections (Feng et al., 2021). Furthermore, recent research by Mabanglo et al. has identified acyldepsipeptides as a novel class of antibiotics that exhibit bactericidal effects against ClpP targets. Consequently, the application of acyldepsipeptides holds promise for effectively treating recalcitrant infections caused by *Enterococcus faecalis* biofilms (Mabanglo et al., 2019).

Sortase A is considered a pivotal enzyme for the survival of *Enterococcus faecalis*, making it a significant therapeutic target in the context of *Enterococcus faecalis* infections. This enzyme plays a substantial role in cross-linking bacterial cell wall proteins by catalyzing the connection between surface proteins and cell wall peptidoglycan, thereby facilitating biofilm formation (Cascioferro et al., 2014). The close association of Sortase A with the initial stages of biofilm formation in *Enterococcus faecalis* has been demonstrated, and its absence profoundly impacts the attachment phase, leading to inadequate biofilm formation (Guiton et al., 2009). Studies have shown that berberine hydrochloride effectively inhibits *Enterococcus faecalis* biofilm formation by suppressing mRNA expression associated with Sortase A and EPS (Chen et al., 2016).

#### 4.1.2 Modulating bacterial communication and signaling pathways to inhibit *Enterococcus faecalis* biofilm formation

QS is a recognized bacterial communication system that facilitates inter-bacterial communication through the production and detection of signaling molecules known as autoinducers (Ramakrishnan et al., 2022). Upon reaching a certain threshold, these molecules trigger alterations in bacterial behavior, including modulation of EPS production, bacterial surface adhesion, expression of virulence factors, and subsequent biofilm formation (Shao et al., 2007). Targeting bacterial QS presents a promising avenue for combating *Enterococcus faecalis* biofilms. The QS inhibitors may target QS at various stages, including signal biosynthesis, degradation of signal molecules, competition for signals binding to cognate receptors, and repression of QS-mediated gene transcription (Kalia et al., 2023).

TC inhibits QS systems and modulates the expression of genes associated with biofilm formation (Vasconcelos et al., 2018). Inhibition of QS has been demonstrated to reduce bacterial colonization and degradation of host tissue components by impeding the production of hydrolytic enzymes, including proteases and gelatinases (Seleem et al., 2020). Moreover, it effectively disrupts bacterial biofilm formation and maturation through interference with the production, perception, or transmission of signaling molecules (Ramakrishnan et al., 2022). Akshaya et al. discovered that TC effectively inhibits the gelatinase activity of *Enterococcus faecalis* by impeding QS, thereby restraining *Enterococcus faecalis* biofilm formation (Akshaya et al., 2023).

Autoinducer-2 (AI-2) functions as a key signaling molecule that facilitates inter-bacterial communication, with its synthesis relying on the LuxS gene. When the bacterial population density reaches a critical level, this signaling molecule AI-2 is released into the surrounding environment, triggering QS behavior among bacteria. Knocking out the LuxS gene modulates AI-2 expression, leading to a consequential impact on *Enterococcus faecalis* biofilm formation. Specifically, lack of LuxS gene expression reduces biofilm size and discreteness while also causing deficiencies in secretions and fibrous connections, thereby significantly affecting *Enterococcus faecalis*’ capability for biofilm formation (Yang et al., 2018). Therefore, insights gained from AI-2 signaling molecules offer valuable information for combating *Enterococcus faecalis* biofilms in the future. One potential approach involves inhibiting the ability of *Enterococcus faecalis* to form biofilms by disrupting the synthesis or transportation of AI-2 signaling molecules.

The role of the Fsr QS system in synthesizing the enzyme gelatinase is crucial for biofilm formation, as gelatinase activity has been reported as one of the initial steps in biofilm generation (Hancock and Perego, 2004a). Cinnamaldehyde reduces biofilm formation by modulating gene expression within the Fsr QS system of *Enterococcus faecalis* (Akshaya et al., 2023). The inhibition of gelatinase production by QS inhibitors is considered essential in preventing degradation of host tissue components caused by bacterial colonization (Seleem et al., 2020). Specifically, cinnamaldehyde significantly decreases the expression of fsrB and fsrC genes within *Enterococcus faecalis* biofilms. This downregulation subsequently reduces response regulator phosphorylation, leading to inhibition of gelE-sprE operon transcription. Consequently, this suppression further attenuates gelatinase activity, ultimately impeding biofilm formation (Nakayama et al., 2006). There is potential for α-pinene found in tea tree oil to hinder *Enterococcus faecalis* biofilm formation by potentially interfering with QS systems (Kerekes et al., 2013). Additionally, probiotics can also exert anti-biofilm activity by disrupting QS via gene regulation (Kumar et al., 2021).

The two-component signal transduction system (TCS) in bacteria, which functions alongside the QS system to sense and respond to environmental signals, plays a crucial role in regulating various bacterial physiological processes, including biofilm formation and maturation, by modulating gene expression and enzyme activity (Zhang et al., 2023). *Enterococcus faecalis* harbors a total of 17 two-component signal transduction systems, with walRK identified as the singular essential TCS that is crucial for determining the viability of the strain (Hancock and Perego, 2004b). WalRK in *Enterococcus faecalis* demonstrates regulatory control over diverse metabolic processes encompassing cell wall synthesis, osmoprotection, and biofilm formation. Consequently, WalRK has been recognized as a pivotal target in combating *Enterococcus faecalis* biofilms (Zhang et al., 2023). By interfering with the auto-phosphorylation process of WalK, it effectively disrupts the functionality of the WalRK system mitigating *Enterococcus faecalis* biofilm formation.

### 4.2 Degradation of structural constituents of mature biofilms

Biofilms, which are characterized by their composition of polysaccharides, proteins, lipids, and eDNA collectively referred to as EPS (Fleming and Rumbaugh, 2017), can be effectively targeted for degradation through the specific breakdown of structural components such as proteins, eDNA, and polysaccharides.

The eDNA framework within EPS is considered essential (Okshevsky and Meyer, 2015). It has been demonstrated that eDNA plays a pivotal role in the formation, structural integrity, and maturation of *Enterococcus faecalis* biofilms (Thomas et al., 2008). Yu et al. employed deoxyribonuclease (DNase) as an enzymatic agent to effectively degrade eDNA molecules, thereby impeding their activity in both *Enterococcus faecalis* cells and biofilms. This intervention significantly reduced the stability of the biofilms. Furthermore, they found that inhibiting eDNA heightened the susceptibility of *Enterococcus faecalis* biofilms to NaOCl treatment (Yu et al., 2019). In previous studies, it has been demonstrated that silver nanoparticles and graphene oxide have the ability to induce oxidative stress, leading to the generation of ROS (Chernousova and Epple, 2013; Martini et al., 2020). These ROS molecules can effectively interact with proteins and eDNA within the extracellular polymeric substances (EPS), thereby disrupting biofilm structures (Ramakrishnan et al., 2022).

Additionally, the phage-derived polysaccharide depolymerase demonstrates promising potential in effectively disrupting biofilm architecture by specifically targeting bacterial capsular polysaccharides and degrading the EPS matrix (Donlan, 2009; Khalifa et al., 2016).

The investigation elucidating the underlying mechanisms of anti-*Enterococcus faecalis* biofilms described above will make a significant contribution to the development of safer and more efficacious agents targeting persistent infections in root canals caused by *Enterococcus faecalis* biofilms.

## 5 Conclusions

*Enterococcus faecalis* is a major culprit in root canal treatment failures, and its biofilm formation significantly enhances antibiotic resistance. These bacteria exhibit survival capabilities under adverse environmental conditions. Elimination of microbes from the pulpal tissue as well as in root canals is the main goal to prevent and treat pulpal and periapical lesions. The persistence of *Enterococcus faecalis* in root canal infections presents significant challenges due to its robust biofilm-forming capabilities and resistance to conventional treatments. Clinically, the current focus of interest lies in the elimination of *Enterococcus faecalis* through the employment of mechanical instrumentation and irrigating solutions with potent antibacterial activity such as NaOCl and CHX. It has been demonstrated that mechanical preparation alone cannot predictably eliminate the bacteria from infected root canals. Current research on various irrigating solutions focuses on investigating their antimicrobial properties, which are further enhanced through the incorporation of surfactants, heating techniques, as well as ultrasonic or sonic agitation. However, these methods have limitations in completely eradicating *Enterococcus faecalis* biofilms. Furthermore, the most common intracanal medicaments, water-based Ca(OH)_2_, exhibit limited efficacy in eradicating bacteria residing within biofilms. Antibiotics have been used as intracanal medication in root canal treatment since at least the 1950s. The triple antibiotic paste prepared by mixing ciprofloxacin, minocycline, and metronidazole with sterile distilled water has been recommended for the management of teeth with incomplete root formation. However, the local application of conventional antibiotics such as chloramphenicol and tetracycline in the root canal space has been restricted due to the high microbial diversity of the intracanal bacterial population and the risks of adverse effects (Ordinola-Zapata et al., 2022). This review highlights several promising strategies to combat *Enterococcus faecalis* biofilms, including nanoparticles, phages, plant derived agents, and probiotics, and also elucidates the underlying mechanisms of targeting *Enterococcus faecalis* biofilms, including inhibiting biofilm formation and effectively disrupting mature or preformed biofilms.

The utilization of nanoparticles, such as metal/metal oxide nanoparticles, propolis nanoparticles, graphene, and its derivatives, in combating *Enterococcus faecalis* biofilm-mediated infections exhibits promising potential for broader therapeutic applications. However, studies on nanoparticles based on *in vitro* research, while providing useful initial insights, often fail to simulate the complexity of real biological systems. They do not adequately account for immune responses or the dynamic conditions of the body, such as blood flow and tissue interactions, leading to potentially misleading results about nanoparticle distribution and efficacy. Moreover, they typically overlook long-term toxicity and accumulation in tissues, highlighting the need for more comprehensive *in vivo* research to understand their real-world safety and effectiveness. Another challenge in achieving eradication of biofilms in root canals lies in addressing *Enterococcus faecalis* biofilms located within anatomically inaccessible regions. The diversity of nanoparticles can be designed to enhance drug aqueous solubility, and by precisely adjusting their chemical compositions, size, surface charge, and other properties, they offer unparalleled flexibility in carrying, retaining, and releasing drugs at precise locations and times (Afkhami et al., 2024).

Phages exhibit a narrower spectrum of inhibition and possess enhanced targeting capabilities in comparison to antibiotics. However, instead of replacing antibiotics, the combination of phages and antibiotics can be superior to the use of single agents. The likely advantages of a combined strategy are enhanced bacterial suppression, more efficient penetration into biofilms, and lowered chances for the emergence of phage resistance. Neutral effects and negative interference between phages and antibiotics have been reported as well. Combined approaches may still be important for at least hampering the development of resistance. In any case, the choice of phage type and antibiotic and their mixing ratios must be given careful consideration when deciding on a dual antibacterial approach (Tagliaferri et al., 2019). Despite facing obstacles and being in its infancy, phage therapy represents a prevailing trend, with phage preparations, phage endolysins, and phage-antibiotic synergies serving as ideal therapeutic agents. On the other hand, the constant rise in strains that are resistant to antibiotics and the negative side effects of chemical irrigants have prompted a search for substitute phytotherapic agents. Numerous phytotherapic agents are showing promise for use as endodontic irrigants because of their antimicrobial, anti-inflammatory, and therapeutic properties. There is little information available regarding the efficiency, safety, and higher quality of these products for use in dentistry. Hence, additional *in vivo* studies must be conducted to confirm these findings. Moreover, the clinical application of combination therapy involving anti-biofilm agents and antimicrobial drugs shows significant promise in managing bacterial biofilm infections. It is imperative to comprehensively explore key attributes of new anti-biofilm agents, including their molecular structure, mechanisms of action, and potential for drug resistance. Future research should focus on elucidating the molecular mechanisms underlying biofilm resistance, optimizing the application of novel anti-biofilm agents, and ensuring their safety and efficacy in clinical settings. A multidisciplinary approach combining traditional and innovative therapies holds the greatest promise for effectively managing persistent endodontic infections caused by *Enterococcus faecalis* biofilms, ultimately improving clinical outcomes and reducing treatment failures.

## Author contributions

SY: Conceptualization, Project administration, Supervision, Visualization, Writing – original draft, Writing – review & editing. XM: Conceptualization, Funding acquisition, Project administration, Supervision, Writing – original draft, Writing – review & editing. YZ: Supervision, Visualization, Writing – review & editing. QB: Project administration, Supervision, Writing – review & editing. YW: Conceptualization, Visualization, Writing – review & editing. XJ: Conceptualization, Visualization, Writing – review & editing. ZX: Project administration, Supervision, Writing – review & editing.
